# The Impact of Upward Social Comparison on Social Media on Appearance Anxiety: A Moderated Mediation Model

**DOI:** 10.3390/bs15010008

**Published:** 2024-12-26

**Authors:** Jinrui Tian, Boxuan Li, Ronghua Zhang

**Affiliations:** Institute of Developmental and Educational Psychology, School of Marxism, Wuhan University, Wuhan 430072, China; 2023201180132@whu.edu.cn (J.T.); 2023201180126@whu.edu.cn (B.L.)

**Keywords:** appearance anxiety, social media, upward social comparison, self-compassion, self-objectification

## Abstract

In the digital age, social media has not only transformed the way individuals interact but has also become a significant platform for self-presentation, especially among young people. Social comparison regarding appearance has become more prevalent in this environment, raising concerns about its impact on mental health. This study specifically examines the effects of upward social comparison (USC) on appearance anxiety, providing empirical support for the relationship between USC and appearance anxiety. Based on the Stress-Vulnerability Model, the Stress and Coping Model, Objectification Theory, and Self-Compassion Theory, the study constructs a moderated mediation model. An anonymous survey was conducted of 397 young adults (mean age = 21.6 years, *SD* = 2.12 years). The results showed that: (1) USC significantly predicted appearance anxiety (*β* = 0.546, *p* < 0.001); (2) self-objectification partially mediated the relationship between USC and appearance anxiety, with the mediation effect accounting for 21% of the total effect; (3) self-compassion moderated the relationship between USC and self-objectification, such that higher levels of self-compassion weakened the effect of USC on self-objectification.

## 1. Introduction

The rapid growth of social media, particularly visually driven platforms like Instagram and TikTok, has significantly amplified social comparisons in areas such as appearance, lifestyle, and achievements (Vogel et al., 2014). On these platforms, upward social comparison—particularly comparing oneself to those in more advantageous situations—has become a prevalent phenomenon in social media use (Festinger, 1954). Individuals are frequently exposed to edited or idealized images that encourage appearance-based social comparisons (Tiggemann et al., 2018). Studies show that upward comparisons are not only widespread on social media, but they also have a significant impact on psychological health, particularly increasing appearance anxiety (Fardouly et al., 2015). Furthermore, these upward comparison images may lead to feelings of inferiority, anxiety, or depression, with this effect being particularly pronounced among adolescents and young women (Cohen et al., 2017).

Upward social comparisons do not always lead to negative outcomes. In some cases, they can motivate individuals to set higher goals (Tiggemann & Slater, 2014). The emotional response often depends on how individuals perceive those they compare themselves to and their coping strategies (Park et al., 2021). Additionally, upward comparisons can influence social media behavior, such as reducing usage or posting negative comments, reflecting the complex psychological dynamics at play.

Self-objectification, often linked to upward social comparisons, tends to increase body dissatisfaction and the tendency to evaluate oneself based on appearance, particularly in response to idealized images on social media (Feltman & Szymanski, 2018). However, Murnen and Seabrook (2012) suggested that self-objectification can also lead to self-reflection and cognitive reappraisal, shifting the focus from external appearance to internal values.

In recent years, individuals’ values have shifted from external standards to internal self-realization and growth (Delle Fave et al., 2011). This change suggests that upward comparisons on social media may not only lead to appearance anxiety but also inspire self-development, driving personal growth (Gonzales & Hancock, 2011). Noll and Frederickson (1998) further noted that self-objectification may evolve from “objectification” to “humanization,” reducing appearance anxiety and fostering self-improvement.

Given the complex effects of upward social comparison and self-objectification, this study aims to examine whether upward comparisons influence appearance anxiety through self-objectification, particularly in social media contexts. Upward social comparison involves comparing oneself to individuals perceived as better off, which can motivate self-improvement but may also lead to feelings of inadequacy (Wood, 1989). Conversely, downward social comparison entails comparing oneself to those perceived as worse off, which may alleviate appearance anxiety by highlighting personal advantages relative to others and enhancing self-esteem (Taylor & Lobel, 1989; Buunk & Gibbons, 2007). This study will also explore the duality of self-objectification, which may both cause anxiety and promote self-improvement through cognitive adjustments. Additionally, the study will consider the potential role of downward social comparison in mitigating appearance anxiety by providing emotional comfort and reinforcing a positive self-concept (Mussweiler & Strack, 2000). Utilizing the Stress-Vulnerability Model (Zubin & Spring, 1977), the Stress and Coping Model (Lazarus & Folkman, 1984), and Self-Compassion Theory (Neff, 2003), this study will analyze the relationships between social comparison, self-objectification, and appearance anxiety.

### 1.1. Conceptualizing the Relationship Between USC and Appearance Anxiety

Social comparison is a fundamental psychological process that can be divided into two main types: upward social comparison and downward social comparison (Buunk & Gibbons, 2007). Upward social comparison involves comparing oneself to individuals perceived as better in certain aspects, which can motivate self-improvement but may also lead to feelings of inadequacy (Wood, 1989). In contrast, downward social comparison entails comparing oneself to those perceived as worse off, which can enhance self-esteem and provide a sense of relief or superiority (Taylor & Lobel, 1989). By distinguishing between these two types of comparisons, we can better understand their unique effects on individuals’ self-perception and behavior (Mussweiler & Strack, 2000). This study will primarily focus on upward social comparison while acknowledging the role of downward social comparison in the broader context of social comparison processes.

In the context of social media, this has evolved into upward social comparison (USC), where people compare themselves to those perceived as better off, especially in appearance and lifestyle (Vogel et al., 2014). Social media, filled with idealized “highlight moments”, encourages users to post flattering images, prompting unconscious comparisons. These comparisons can lead to negative self-evaluations, contributing to psychological issues like appearance anxiety and low self-esteem (Fardouly et al., 2015).

Research highlights that young adults are particularly vulnerable to the effects of USC. Tiggemann & Slater (2014) observed that young adults who frequently engage with social media are more prone to experience heightened dissatisfaction with their appearance as a result of these comparisons. Moreover, Kim and Chock (2015) found that individuals who are highly appearance-conscious may seek self-identity through appearance-focused content on social media, thus entering a negative cycle that exacerbates appearance anxiety (Bue, 2020).

USC impacts not only female users in terms of appearance anxiety but also affects male users, particularly concerning body shape and muscle mass. Studies such as those by Ridgway and Clayton (2016) reveal that men may experience increased dissatisfaction with their bodies after viewing idealized muscular images, sometimes even leading to considerations of cosmetic surgery (De Vries et al., 2014). Thus, the effects of USC transcend gender, indicating its widespread impact on appearance anxiety.

Cultural factors also modulate the effects of USC. In collectivist cultures, for example, USC may bolster social support systems by leveraging the successes of others, thus promoting group identity and positive development (Kim & Markus, 1999). This highlights the role of upward comparison not only in affecting individual psychology but also in shaping social interactions.

Recent studies by Wu et al. (2024) highlight the dual impact of social media on appearance anxiety. Idealized images can increase anxiety, but platforms promoting diverse esthetics and authenticity, like the “True Beauty” movement, can reduce it. Social Media Literacy (SML) also serves as a protective factor, with Scully et al. (2023) finding that adolescents with higher SML are better at critically evaluating content, which helps reduce appearance anxiety by resisting unrealistic beauty standards.

In conclusion, USC is a prevalent phenomenon on social media, especially in appearance-related comparisons, and exerts a considerable influence on individual psychology. Frequent upward comparisons can exacerbate appearance anxiety, lead to self-objectification, and perpetuate a cycle of negative emotions. However, USC can also act as a catalyst for personal growth and self-improvement in certain contexts. Understanding the dual impacts of USC is thus of paramount importance, leading to the following hypothesis:

**H1.**  *Upward social comparison on social media will positively influence the degree of appearance anxiety.*

### 1.2. Conceptualizing the Mediating Role of Self-Objectification

Self-objectification, a core concept in objectification theory, suggests that societal expectations and media portrayals of women’s bodies lead them to view themselves as objects evaluated by appearance (Fredrickson & Roberts, 1997). This process significantly affects mental health, contributing to issues like appearance anxiety, disordered eating, and depression (Tiggemann & Williams, 2011). Research supports the idea that self-objectification drives body dissatisfaction, as individuals internalize societal beauty standards and base their worth on appearance (Calogero et al., 2005). The theory also highlights the intersection between gendered violence and self-objectification, particularly when victimization intensifies these societal pressures (Fredrickson & Roberts, 1997). Media portrayals of women amplify objectifying norms, leading to widespread self-objectification and increased body dissatisfaction (Levine & Murnen, 2009).

Media exposure, especially to idealized body images, plays a key role in promoting body image disturbances and disordered eating, reinforcing the harmful psychological effects of objectification (Grabe et al., 2008). On social media, idealized body images are often presented through curated and edited photos, leading individuals to focus excessively on their appearance and increasing self-objectification (Tiggemann & Slater, 2014). This effect is more pronounced in social comparison contexts. For example, Karsay et al. (2018) found that compared to traditional media, social media images and videos are more likely to reinforce self-objectification due to more opportunities for appearance-based comparisons.

This phenomenon is not limited to women; men also face similar societal pressures. Research by Rollero and De Piccoli (2017) further confirms that with the rise of fitness culture, men are more inclined to display strength and muscle, which is closely related to the concept of objectification. In contrast, women are more likely to internalize slim and graceful body ideals (Tiggemann & Slater, 2014). These gendered body standards not only bring about gender-specific patterns of self-objectification but also influence individuals’ perceptions and evaluations of their own body image.

The Stress-Vulnerability Model (Zubin & Spring, 1977) provides a framework for understanding how self-objectification contributes to appearance anxiety. When individuals face external pressures, a lack of psychological resilience makes them more vulnerable to appearance anxiety. As a vulnerability factor, self-objectification amplifies this effect, making individuals more likely to link their self-worth to appearance. The long-term impact of objectification extends beyond anxiety and body dissatisfaction, also impairing cognitive function. Winn and Cornelius (2020) found that self-objectification depletes cognitive resources, such as attention and working memory, weakening performance in other tasks and further harming mental health.

Building on previous studies, on social media, USC is often linked to idealized body images, leading individuals to focus more on their appearance and increasing self-objectification. Therefore, this study hypothesizes that self-objectification mediates the link between USC and appearance anxiety, as individuals internalize external pressures through self-objectification, ultimately resulting in appearance anxiety.

**H2.**  *Self-objectification mediates the relationship between USC and appearance anxiety on social media.*

### 1.3. Conceptualizing the Moderating Role of Self-Compassion

The Stress and Coping Model (Lazarus & Folkman, 1984) explains how individuals respond to stressors, with primary appraisal assessing threat and secondary appraisal evaluating coping resources. Self-compassion, a key resource, helps regulate negative emotions (Neff & Germer, 2022). Higher self-compassion levels enable individuals to mitigate negative emotions, especially during stressors like upward social comparisons tied to appearance anxiety. Self-compassion, consisting of self-kindness, common humanity, and mindfulness, is crucial for managing emotional distress (Neff, 2003). Recent studies show that self-compassion not only improves emotional states but also buffers the link between upward social comparisons and appearance anxiety, particularly in competitive contexts (Neff & Germer, 2013a). Furthermore, cultural differences influence self-compassion expression, with collectivist cultures emphasizing responsibility and humility, and individualistic cultures focusing on self-care and acceptance (Neff et al., 2008; Wong & Mak, 2013).

Self-kindness, a key component of self-compassion, encourages individuals to treat themselves with care during setbacks rather than harshly criticizing themselves (Neff, 2003). Consistent with the stress and coping model, self-kindness enhances coping abilities in the secondary appraisal stage, reducing self-criticism during appearance-related anxiety and alleviating negative emotions from appearance comparisons (Neff & Pommier, 2013). Homan and Tylka (2015) found that self-kindness not only reduces appearance concerns but also promotes positive self-evaluations, improving mental well-being.

Common humanity, another facet of self-compassion, views personal suffering and imperfections as shared human experiences, which helps individuals reframe stressors and reduce the sense of isolation caused by social comparisons, thus lowering appearance anxiety (Neff, 2003). Research has shown that that common humanity alleviates loneliness and appearance anxiety by fostering a more accepting view of one’s appearance (Leary et al., 2007).

Mindfulness, the third element of self-compassion, involves non-judgmental acceptance of emotional experiences, which helps manage stress by preventing excessive emotional reactions or avoidance (Neff, 2003). According to the Stress and Coping Model, mindfulness enhances emotional awareness during stressors, especially in social contexts, helping individuals manage appearance-related anxiety by reducing undue focus on appearance (Braun et al., 2016; Diedrich et al., 2014).

Self-compassion not only aids in emotional regulation but may also moderate the relationship between self-objectification and appearance anxiety. Self-objectification, which involves viewing oneself as an object for external evaluation, often leads to negative self-perception, internalized shame, and heightened anxiety. Research consistently identifies self-objectification as a key predictor of appearance anxiety (Moradi & Huang, 2008). In this context, self-compassion plays a pivotal role by moderating the negative effects of self-objectification on mental health. Specifically, self-compassion encourages individuals to approach themselves with kindness and acceptance rather than self-criticism, reducing the internalization of appearance-based evaluations and thus mitigating appearance anxiety.

Studies have demonstrated that self-compassion can mitigate the negative effects of self-objectification, including appearance anxiety. For instance, Gao et al. (2023) found that self-compassion alleviates appearance anxiety induced by self-objectification by reducing individuals’ reliance on external appearance evaluations, particularly on social media. These findings suggest that individuals with higher levels of self-compassion are better able to cope with the pressures of appearance-based comparisons and are less likely to internalize these pressures as anxiety. Building on these insights, this study proposes the following hypotheses:

**H3.**  *Self-compassion moderates the relationship between upward social comparison (USC) and self-objectification, such that higher levels of self-compassion will weaken the negative impact of USC on self-objectification.*

**H4.**  *The three dimensions of self-compassion—self-kindness (including inverse scoring of self-criticism), common humanity (including inverse scoring of loneliness), and mindfulness (including inverse scoring of over-absorption)—will act as moderators in the relationship between USC and appearance anxiety. Self-compassion will moderate the mediating role of self-objectification between USC and appearance anxiety.*

### 1.4. The Present Study

The pervasive use of social media has intensified upward social comparisons (USC), particularly concerning appearance, often triggering appearance anxiety exacerbated by self-objectification (Tiggemann & Slater, 2014; Fardouly et al., 2015). While the relationship between USC and appearance anxiety is well-documented, research on the interplay between USC, self-objectification, and self-compassion remains sparse (Tiggemann & Slater, 2014; Neff, 2003). Self-objectification, often triggered by USC, involves perceiving oneself as an object for external evaluation, amplifying appearance anxiety (Fredrickson & Roberts, 1997). Conversely, self-compassion can mitigate these negative effects by promoting gentler self-perception through self-kindness, common humanity, and mindfulness, reducing appearance anxiety (Neff, 2003; Neff & Germer, 2013b). These dimensions help individuals navigate and alleviate pressures from social comparisons (Neff, 2003). However, detailed research on how these variables interact, particularly through self-compassion’s mechanisms, is lacking (Tiggemann & Slater, 2014).

This highlights the need for a more comprehensive examination of USC, self-objectification, and self-compassion, especially within different cultural contexts. In China, influenced by collectivism and a culture of humility, self-compassion typically emphasizes individual responsibility towards both oneself and others. Excessive self-pity may be viewed as self-indulgence, which contrasts with the Western focus on individualism and self-care (Koopmann-Holm et al., 2024). Thus, understanding these cultural differences is crucial for exploring self-compassion’s role in mitigating appearance anxiety across cultural backgrounds.

This study, based on the Stress-Vulnerability Model (Zubin & Spring, 1977), the Stress and Coping Model (Lazarus & Folkman, 1984), and Neff’s (2003) theory of self-compassion, hypothesizes that self-objectification mediates the relationship between upward social comparison (USC) and appearance anxiety, with self-compassion moderating this relationship to alleviate its negative effects. The research integrates these theories with a focus on Chinese young adults, exploring the cultural differences in self-compassion between collectivist and Western individualistic contexts, aiming to enhance the cultural applicability of these constructs and provide a theoretical basis for fostering self-compassion. Through this exploration, this study aims to deepen the understanding of youth appearance anxiety and lay a foundation for future intervention strategies.

## 2. Method

### 2.1. Participants

This study recruited a total of 397 undergraduate students, with 44.33% male and 55.67% female participants, ranging in age from 18 to 25 years, with a mean age of 21.6 years (*SD* = 2.13). Among them, 57.9% were from urban areas and 42.1% from rural areas. The initial survey was completed by 443 participants, but after applying the exclusion criteria, a total of 397 participants remained in the analysis, with exclusions made for the following reasons: 5 participants were excluded for incorrect age, 10 participants for not using social media regularly, and 31 participants for not completing the survey.

Participants were asked to report their average daily social media usage time, the platforms they used most frequently (e.g., WeChat, TikTok, Instagram, Facebook), and the primary activities they engaged in on these platforms (e.g., browsing, posting, social interaction). The questionnaires were presented in the following order: first, participants provided demographic information, including age, gender, location (urban vs. rural), and social media usage details. Following this, they completed the main survey questions related to social comparison, self-objectification, and appearance anxiety.

Data collection took place from December 2023 to January 2024, with participants recruited through email for online participation and posters in classrooms for offline participation. A total of 198 students completed the questionnaire in class by scanning a QR code, while the remaining participants completed it online. Over 60% of the participants reported using social media for more than three hours per day, indicating a significant influence of social media on their thoughts and behaviors. All participants signed an informed consent form before completing the questionnaire, and participation was voluntary. Participants were also offered a small reward of sticky notes as an incentive for their participation.

### 2.2. Measures

After reading the instructions, participants completed a series of questionnaires designed to measure the key variables in this study. The questionnaires were distributed both online via the Wenjuanxing platform and offline, allowing participants to respond to each item based on their personal experiences and perceptions.

#### 2.2.1. Youth Appearance Anxiety Scale

In this study, the Youth Appearance Anxiety Scale revised by Luo et al. (2023) was used to measure appearance anxiety. The questionnaire consists of 25 items covering four aspects: appearance anxiety, body anxiety, skin anxiety, and behavioral investment, e.g., “I always spend a lot of time and energy covering up my appearance flaws (e.g., makeup)”. Participants were asked to report the degree of agreement with each item based on their actual situation. The questionnaire uses a seven-point rating scale, ranging from “strongly disagree” (1) to “strongly agree” (7), with higher scores indicating stronger appearance anxiety. The Cronbach’s alpha coefficient for this questionnaire in this study is 0.97.

#### 2.2.2. Upward Social Comparison Scale

This scale, developed by Gibbons and Buunk (1999), uses the upward comparison subscale. Following previous research, the comparison context was limited to “social networking” scenarios. This questionnaire, being concise and easy to administer, contains six items, e. g., “When using social media, I often like to compare myself with those who are better off than me”. It employs a 5-point Likert scale, from “strongly disagree” (1) to “strongly agree” (5). Higher individual scores indicate a greater tendency for upward social comparison on social networks. The Cronbach’s alpha coefficient for this questionnaire in this study is 0.94.

#### 2.2.3. Self-Compassion Scale (SCS)

The SCS, developed by Neff (2003), consists of 26 items divided into six subscales: Self-Kindness (5 items), Self-Judgment (5 items), Common Humanity (4 items), Isolation (4 items), Mindfulness (4 items), and Over-identification (4 items), e.g., “I’m disapproving and judgmental about my own flaws and inadequacies”. Items are rated on a scale from 1 (“does not fit at all”) to 5 (“fits very well”), with 9 items scored in reverse. The Cronbach’s alpha coefficient for this questionnaire in this study is 0.88.

#### 2.2.4. Chinese Version of the Objectified Body Consciousness Scale Body Surveillance Subscale

This scale, developed by (McKinley & Hyde, 1996), includes 24 items specifically divided into three dimensions: body surveillance, body shame, and control beliefs. The body surveillance dimension, considered a significant manifestation of self-objectification, is widely used as a measure of self-objectification, assessing how a person monitors their body and views it as an outsider. It was localized and revised by (Chen & Jiang, 2007) e.g., “During the day, I think about how I look many times”. It has 8 items and uses a 5-point Likert scale, from “completely disagree” to “completely agree”. The Cronbach’s alpha coefficient for this questionnaire in this study is 0.86.

### 2.3. Analysis Method

This study employed SPSS 26.0 software for data analysis. Initially, independent sample *t*-tests were used to evaluate gender differences in the sample. Subsequently, Pearson correlation analysis was conducted to explore the relationships between key variables: upward social comparison, self-objectification, self-compassion, and appearance anxiety.

To examine the mediating role of self-objectification in the relationship between upward social comparison and appearance anxiety, as well as to assess the moderating effect of self-compassion on this mediation, Hayes’s moderation-mediation analysis (PROCESS Model 7) was utilized. This approach was specifically chosen to analyze whether self-compassion modifies the pathway through which upward social comparison impacts appearance anxiety via self-objectification. The significance of the moderated mediation effect was determined using the bootstrap method, with 5000 bootstrap samples for calculating 95% confidence intervals. An effect was deemed statistically significant if the confidence interval did not include zero.

Further, to elucidate the specifics of the moderation effect, a simple slopes analysis was performed. This analysis aimed to investigate the effect of upward social comparison on appearance anxiety at varying levels of self-compassion. Such analysis helps to demonstrate how the relationship between social comparison and anxiety changes based on individual levels of self-compassion, providing deeper insights into the potential of self-compassion to mitigate the adverse effects of upward social comparisons.

## 3. Result

### 3.1. Common Method Bias

In psychological research, common method bias (CMB) often arises as a prominent issue due to the use of self-reported data. To mitigate the potential impact of this issue, we have implemented several measures, including collecting data in batches, utilizing reverse scoring to reduce response pattern bias, and ensuring the anonymity of responses to lower social desirability bias. By conducting Harman’s single-factor test, we identified nine factors with eigenvalues greater than 1. However, the first factor only explained 27.48% of the variance, well below the critical threshold of 40%. This indicates that common method bias does not pose a severe problem in our dataset. These measures ensure the reliability of the study, providing significant methodological support for the use of self-reported data.

### 3.2. Participant Characteristics and Correlation Matrix

Table 1 shows that appearance anxiety is significantly positively correlated with upward social comparison (r = 0.546, *p* < 0.01), indicating that higher levels of upward social comparison are linked to greater appearance anxiety. A positive correlation between appearance anxiety and self-objectification (r = 0.497, *p* < 0.01) suggests that lower appearance anxiety is associated with reduced self-objectification. Self-compassion is significantly negatively correlated with appearance anxiety (r = −0.354, *p* < 0.01), indicating that individuals with higher levels of self-compassion tend to exhibit lower levels of appearance anxiety. The dimensions of self-compassion (self-kindness, common humanity, and mindfulness) are also negatively correlated with appearance anxiety, with the correlation weakening from self-kindness to mindfulness. According to the results of the independent samples *t*-test, it shows that women experiencing higher levels of appearance anxiety than men (*t* = −1.187, *p* < 0.05). Women also report higher levels of upward social comparison than men (*t* = −0.231, *p* < 0.05). Additionally, the study analyzed gender differences in self-compassion and self-objectification. The results of the independent samples *t*-test indicated that the differences in self-compassion (*t* = −0.05, *p* > 0.05) and self-objectification (*t* = −0.07, *p* > 0.05) between men and women were not statistically significant. These findings suggest that although self-compassion and self-objectification theoretically have significant impacts on individual psychological health and social behavior, gender does not significantly influence these variables in our sample.

### 3.3. The Relationship Between USC and Appearance Anxiety

As shown in Table 2, the analysis confirmed that upward social comparison (USC) is a strong predictor of appearance anxiety, yielding significant statistical results (B = 1.889, *t* = 6.483, *p* < 0.001). Further analysis highlighted the mediating role of self-objectification; specifically, USC significantly predicted self-objectification (B = 0.723, *t* = 2.324, *p* < 0.001), which, in turn, had a strong predictive effect on appearance anxiety (B = 0.748, *t* = 8.059, *p* < 0.001). These results not only emphasize the direct link between USC and appearance anxiety but also underscore the critical mediating role of self-objectification within this relationship. The overall results indicate that the total effect of USC on appearance anxiety is 0.855, highlighting a strong positive relationship between these variables. The direct effect of USC on appearance anxiety is 0.669, underscoring that USC significantly influences appearance anxiety independently of any mediating variables. The indirect effect through self-objectification is 0.186, with a 95% bootstrap confidence interval ranging from 0.120 to 0.256. This significant indirect effect confirms the pivotal mediating role of self-objectification between USC and appearance anxiety. These results validate Hypothesis 2, suggesting that self-objectification serves as a mediator between USC and appearance anxiety.

**Figure 1:**
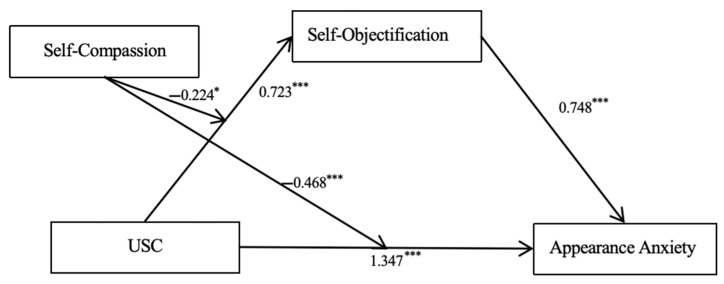
The moderated mediation model of USC, Self-Objectification, Self-Compassion, and appearance anxiety. *Note.* * *p* < 0.05, *** *p* < 0.001.

The analysis investigated the moderating role of self-compassion within the mediation model, exploring its impact on both the direct pathway from upward social comparison (USC) to appearance anxiety and the mediated pathway through self-objectification. As illustrated in Table 2, the findings reveal that self-compassion significantly reduces the direct negative impact of USC on appearance anxiety, with higher levels of self-compassion associated with a lesser degree of appearance anxiety (Interaction term USC × Self-Compassion for direct effect: B = −0.468, *t* = −4.039, *p* < 0.001). Additionally, self-compassion also moderates the mediated impact through self-objectification, effectively diminishing the influence of USC on appearance anxiety via self-objectification (Interaction term USC × Self-Compassion for mediated effect: B = −0.224, *t* = −3.834, *p* < 0.001). The complete model illustrating these relationships and effects is depicted in Figure 1.

**Figure 2:**
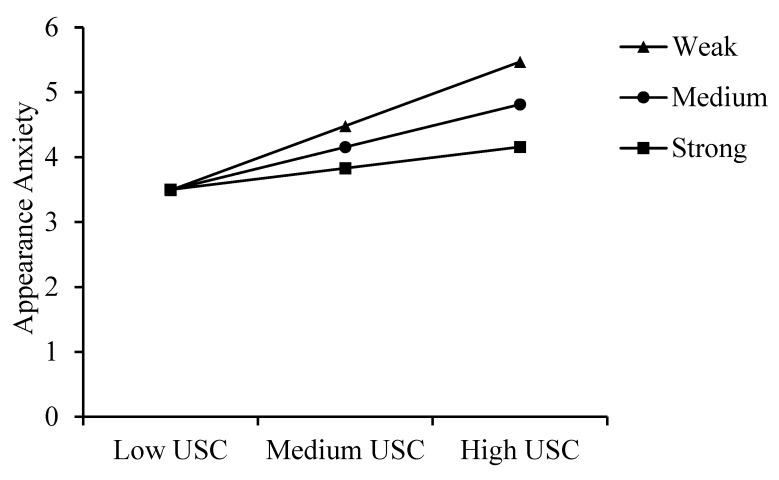
The moderating role of self-compassion between USC and appearance anxiety. *Note. Weak represents low self-compassion. Medium represents average self-compassion. Strong represents high self-compassion.*

**Figure 3:**
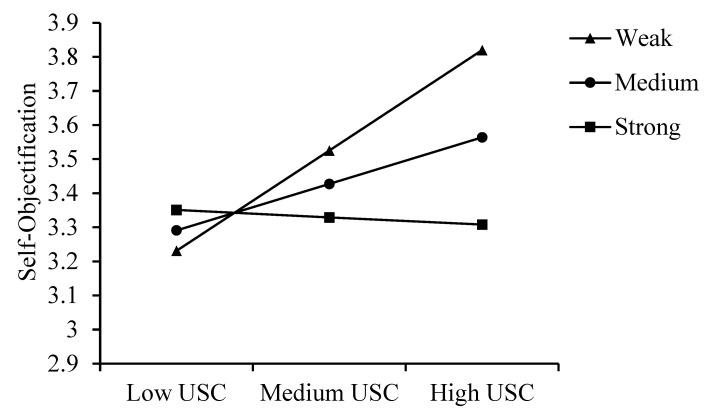
The moderating role of self-compassion between USC and Self-Objectification. *Note. Weak represents low self-compassion. Medium represents average self-compassion. Strong represents high self-compassion.*

Table 3 shows that, at low and mean levels of self-compassion, the indirect effect of upward social comparison (USC) on appearance anxiety through self-objectification is positive and significant, while at high levels of self-compassion, this indirect effect becomes non-significant, suggesting that self-compassion weakens the mediating role of self-objectification. Providing detailed insights into how moderation varies, complementing Figure 2 and Figure 3 for a clearer understanding of the relationships.

### 3.4. Moderated Mediation Models

In the analytical approach, separate moderated mediation models were employed for each of the sub-scales to thoroughly assess the specific impacts and interactions within the dataset. This method allowed for tailored analyses to the nuances of each sub-scale, providing a more detailed and accurate understanding of the underlying dynamics. Detailed descriptions of these models and the rationale for using separate analyses are provided below to ensure transparency and reproducibility of our findings. To verify the distinct moderating roles of the three dimensions of self-compassion—self-kindness (including inverse scoring of self-criticism), common humanity (including inverse scoring of loneliness), and mindfulness (including inverse scoring of over-identification)—in the relationship between USC and appearance anxiety, Model 7 of the PROCESS v3.3 macro was used to test the moderated mediation model. The results indicated that self-kindness significantly moderates the relationship between USC and appearance anxiety (USC × self-kindness: B = −0.215, *t* = −3.747, *p* < 0.001), common humanity significantly moderates the relationship between USC and appearance anxiety (USC × common humanity: B = −0.166, *t* = −3.347, *p* < 0.001), and mindfulness significantly moderates the relationship between USC and appearance anxiety (USC × mindfulness: B = −0.194, *t* = −3.583, *p* < 0.001). These findings reveal that self-kindness, common humanity, and mindfulness each reduce the impact of USC on appearance anxiety in different ways, suggesting that these dimensions can serve as protective factors to help individuals cope with the psychological stress arising from social comparison, supporting Hypothesis 4.

## 4. Discussion

This study investigates the impact of upward social comparisons (USC) on social media on young adults expirancing anxiety, focusing on the moderating and mediating roles of self-compassion and self-objectification. Specifically, it examines the distinct moderating effects of the three dimensions of self-compassion. Survey data from 397 undergraduates reveal that USC on social media significantly increases appearance anxiety, consistent with Fardouly et al. (2015), who found that social media comparisons heighten body dissatisfaction and psychological stress among young adults.

The Stress-Vulnerability and Stress-Coping models provide theoretical support for understanding these dynamics. This research uncovers the internal mechanisms linking USC and appearance anxiety, highlighting self-objectification and self-compassion as mediating factors. By analyzing these conditional effects, the study offers a nuanced understanding of psychological interactions in this study.

### 4.1. The Mediating Role of Self-Objectification

The mediating variable clarifies the process by which upward social comparison (USC) influences appearance anxiety. Consistent with the hypothesis, the results of this study indicate that self-objectification acts as a partial mediator in the relationship between USC and appearance anxiety. This mediating effect accounts for 39% of the total effect, confirming that the interaction between an individual’s internal vulnerabilities and external stressors is a key mechanism in the development of psychological stress and mental health issues, as outlined in the stress-vulnerability model (Zubin & Spring, 1977; Gotlib et al., 2008; Huizink & De Rooij, 2018). Furthermore, self-objectification not only depletes cognitive resources but also biases cognitive schemas towards appearance-related concerns. This not only increases self-conscious emotions such as shame and anxiety but also exacerbates the development of appearance anxiety and psychological stress (Fredrickson & Roberts, 1997; Engeln, 2023). In young adults, a group undergoing rapid psychological and physical changes, these maladaptive patterns are particularly pronounced and closely linked to social anxiety and depressive symptoms (Calvete et al., 2013; Grabe et al., 2007). Therefore, during the developmental years, significant changes occur physiologically, psychologically, and socially. Thus, it is critical to understand the risk factors associated with self-objectification and their impact on mental health.

Social media comparisons can intensify appearance anxiety, consistent with Normative Influence Theory (Cialdini & Goldstein, 2004), which asserts that societal norms and expectations shape individuals’ self-concepts and behaviors. Platforms promoting idealized images may drive individuals to increase self-objectification and anxiety. Significant gender differences are evident; women, more affected by societal emphasis on appearance, report higher levels of anxiety compared to men who may underreport due to traditional masculine norms. Moreover, reducing social media use after upward comparisons can mitigate associated stress and anxiety, suggesting that less engagement could serve as an effective strategy for managing appearance anxiety (Vogel et al., 2014).

### 4.2. The Moderating Role of Self-Compassion

Self-compassion not only enables individuals to deeply understand and accept their limitations but also lays the groundwork for self-growth and improvement. It aids individuals in identifying growth opportunities when faced with challenges, which promotes healthier self-development (Neff, 2011). In this study, it was found that self-compassion significantly moderated the impact of upward social comparison (USC) on self-objectification, which in turn affects appearance anxiety. Specifically, self-compassion was examined for its role in moderating how USC influences self-objectification before this self-perception could impact appearance anxiety, thereby elucidating its protective role in the psychological processes triggered by social comparison. This resonates with the stress-coping model (Lazarus & Folkman, 1984), which posits that when individuals face stressors (such as negative social comparison), internal coping resources (such as self-compassion) can alleviate the negative impact of the stress. Self-compassion reduces the tendency towards self-objectification through supportive self-dialog and emotional regulation, thereby lessening appearance anxiety triggered by social comparison. This process highlights the protective role of self-compassion in moderating psychological adversity and provides a pathway for intervention by enhancing individuals’ self-compassion to cope with psychological stress and anxiety arising from social comparison. Hence, self-compassion emerges as an important protective factor in this study, offering young adults necessary support to buffer the detrimental effects of appearance anxiety.

The findings of this study provide compelling evidence regarding the differential moderating effects of the three dimensions of self-compassion—self-kindness, common humanity, and mindfulness—on the relationship between upward social comparison (USC) and appearance anxiety. Specifically, the dimension of self-kindness exhibited the most potent moderating effect (B = −0.215, *t* = −3.747, *p* < 0.001). This underscores the pivotal role of self-kindness in reducing the detrimental impact of USC on appearance anxiety, highlighting the critical importance of fostering self-acceptance skills in mental health interventions.

Furthermore, the significant moderating effects observed for the common humanity and mindfulness dimensions emphasize the need for interventions that broaden individuals’ perspectives on the universal nature of human experiences and enhance mindfulness skills. Such interventions can equip individuals to approach social comparisons on social media with a more balanced and detached perspective, effectively reducing their adverse psychological impacts.

The results suggest that, while self-compassion indeed influences the relationship between USC and self-objectification, its protective ability is limited in certain conditions, particularly under intense USC situations. This finding suggests that in extreme social comparison environments, self-objectification may become so pronounced that even a strong self-compassion ability may struggle to exert significant protective effects. These data highlight the need to consider the intensity and frequency of social comparison when designing interventions targeting self-objectification. However, it is crucial to emphasize that the suggestions outlined here are proposed specifically for guiding future research endeavors. Further empirical exploration is necessary to delineate under what specific conditions self-compassion can effectively mitigate the psychological stress induced by negative social comparisons. Additionally, research should investigate how psychological interventions can be designed to enhance individuals’ resilience when confronted with extreme negative comparisons. These inquiries are essential for developing more robust theoretical frameworks and practical interventions that address the complexities of social comparison dynamics.

Normative Influence Theory (Cialdini & Goldstein, 2004) highlights that self-compassion can alleviate some of the psychological stress caused by social media comparisons, but its protective effects against appearance anxiety are not absolute. This suggests that while self-compassion serves as a valuable protective factor, it may not fully counteract the risks of appearance anxiety within the pervasive social media environment. Therefore, psychological health interventions for young adults should adopt a dual approach, focusing on enhancing self-compassion alongside strategies to minimize the prevalence and impact of harmful social comparisons. Such interventions can better support mental health and promote the development of a positive self-image. Future research should explore targeted interventions leveraging self-compassion to mitigate appearance anxiety more effectively in social media contexts.

The findings of this study highlight the protective role of self-compassion in reducing the negative effects of upward social comparison on self-objectification and appearance anxiety. Integrating self-compassion into mental health interventions can empower young adults to build resilience and foster a positive self-image, even in the face of social media pressures. By combining individual self-compassion strategies with systemic improvements to social media environments, there is significant potential to not only alleviate appearance-related distress but also enhance overall well-being. These efforts can pave the way for a healthier, more positive future for young adults navigating the challenges of the digital age.

### 4.3. Research Insights and Shortcomings

Although this study provides important insights into the relationship between social media use and young adults’ appearance anxiety, revealing key internal mechanisms, the findings hold significant theoretical implications for understanding the connection between upward social comparison on social media and appearance anxiety in young adults. Moreover, the results offer crucial guidance for the development of prevention and intervention strategies targeting adolescent appearance anxiety.

First, it is essential to emphasize the impact of upward social comparison on social media on adolescent mental health. Such comparisons often trigger self-doubt and anxiety among young adults, and, in some cases, may lead to more serious mental health problems. It is necessary to actively monitor young adults’ daily lives and social media usage to understand the potential effects on their mental health. Additionally, attention should be paid to young adults’ behavioral patterns on social media and their psychological changes to provide timely guidance and support when needed.

Furthermore, parents and educators should remain vigilant and foster awareness among young adults regarding the healthy use of social media. This involves educating young adults to recognize unhealthy social media behaviors and encouraging them to engage in offline activities, helping them to develop a balanced lifestyle in relation to social media use. Schools and communities should offer relevant counseling resources, such as mental health workshops, social skills training, and stress management courses, to assist young adults in building a healthy self-esteem and positive self-image.

Finally, policymakers should consider implementing appropriate regulations to limit targeted social media marketing aimed at young adults and to protect them from negative social media influences. Social media platforms should enforce age-appropriate content policies and monitor advertisements and content to prevent the promotion of harmful beauty standards and unrealistic life portrayals.

However, there are several limitations to the study. It primarily relies on a sample of young adults from a specific region, which may limit the generalizability of the results. Young adults from different cultural backgrounds may exhibit significant differences in their social media usage behaviors and psychological responses. Additionally, the use of a cross-sectional design limits our ability to infer causality. Although validated scales were used, self-reported survey data may be subject to social desirability effects, where participants’ responses might be influenced by concerns about social acceptance, thereby potentially affecting the accuracy of the data.

## 5. Conclusions

(1) Upward social comparison on social media is a significant positive predictor of adolescent appearance anxiety. (2) Self-compassion indirectly affects appearance anxiety through self-objectification, with self-objectification partially mediating the relationship between upward social comparison on social media and appearance anxiety. (3) Self-compassion moderates the relationship between upward social comparison on social media and self-objectification; when self-compassion levels are high, the negative correlation between upward social comparison on social media and self-objectification is significantly strengthened.

## Figures and Tables

**Table 1:** Participant characteristics and correlation matrix.

Variable		1	2	3	4	5	6	7	8
1	Age	—							
2	Appearance Anxiety	0.179 **	—						
3	Upward Social Comparison	0.104 *	0.546 **	—					
4	Self-Objectification	0.096	0.497 **	0.310 **	—				
5	Self-Compassion	−0.123 *	−0.354 **	−0.267 **	−0.241 **	—			
6	Self-Kindness	−0.128 *	−0.319 **	−0.249 **	−0.248 **	0.942 **	—		
7	Common Humanity	−0.108 *	−0.368 **	−0.286 **	−0.218 **	0.912 **	0.780 **	—	
8	Mindfulness Awareness	−0.102 *	−0.297 **	−0.204 **	−0.197 **	0.920 **	0.802 **	0.770 **	—
*M ± SD*		21.6 ± 2.12	4.24 ± 1.68	3.35 ± 1.02	2.62 ± 0.71	3.47 ± 0.75	2.68 ± 0.78	2.6 ± 0.77	2.55 ± 0.75

Note: *N* = 397. Correlation coefficients were obtained using the Bootstrap method. * *p* < 0.05, ** *p* < 0.01.

**Table 2:** Test of a moderated mediation model.

	Appearance Anxiety		Self-Objectification		Appearance Anxiety	
	*β*	*t*	*β*	*t*	*β*	*t*
USC	1.889	6.483 ***	0.723	2.324 ***	1.347	4.840 ***
Self-Compassion	1.137	2.626 **	0.625	4.928 **	0.669	1.650
USC × Self-Compassion	−0.468	−4.039 ***	−0.224	−3.834 ***	−0.300	−2.746 **
Self-Objectification					0.748	8.059 ***
*R* ^2^	0.334		0.142		0.429	
*F*	65.727 ***		21.755 ***		73.553 ***	

*Note.* ** *p* < 0.01, *** *p* < 0.001.

**Table 3:** Indirect effect values at different levels of self-compassion.

Level of Moderating Variable	Indirect Effect Value	BootLLCI	BootULCI
Low Level	0.258	0.157	0.356
Mean Level	0.12	0.057	0.186
High Level	−0.019	−0.126	0.091

## Data Availability

Due to privacy, the datasets involved in this study are not publicly available but are available from the author Jinrui Tian on reasonable request.

## References

[B1-behavsci-15-00008] Braun T. D., Park C. L., Gorin A. (2016). Self-compassion, body image, and disordered eating: A review of the literature. Body Image.

[B2-behavsci-15-00008] Bue A. C. C. (2020). The looking glass selfie: Instagram use frequency predicts visual attention to high-anxiety body regions in young women. Computers in Human Behavior.

[B3-behavsci-15-00008] Buunk B. P., Gibbons F. X. (2007). Social comparison: The end of a theory and the emergence of a field. Organizational Behavior and Human Decision Processes.

[B4-behavsci-15-00008] Calogero R. M., Davis W. N., Thompson J. K. (2005). The role of self-objectification in the experience of women’s body image disturbance: A test of objectification theory. Journal of Social and Clinical Psychology.

[B5-behavsci-15-00008] Calvete E., Orue I., Hankin B. L. (2013). Early maladaptive schemas and social anxiety in adolescents: The mediating role of anxious automatic thoughts. Journal of Anxiety Disorders.

[B6-behavsci-15-00008] Chen X., Jiang Y. -J. (2007). Revision of the College Students’ Body Consciousness Scale. Chinese Journal of Mental Health.

[B7-behavsci-15-00008] Cialdini R. B., Goldstein N. J. (2004). Social influence: Compliance and conformity. Annual Review of Psychology.

[B8-behavsci-15-00008] Cohen R., Newton-John T., Slater A. (2017). The relationship between Facebook and Instagram appearance-focused activities and body image concerns in young women. Body Image.

[B9-behavsci-15-00008] De Vries D. A., Peter J., Nikken P., De Graaf H. (2014). The effect of social network site use on appearance investment and desire for cosmetic surgery among adolescent boys and girls. Sex Roles.

[B10-behavsci-15-00008] Delle Fave A., Brdar I., Freire T., Vella-Brodrick D., Wissing M. P. (2011). The Eudaimonic and Hedonic Components of Happiness: Qualitative and Quantitative Findings. Social Indicators Research.

[B11-behavsci-15-00008] Diedrich A., Grant M., Hofmann S. G., Hiller W., Berking M. (2014). Self-compassion as an emotion regulation strategy in major depressive disorder. Behaviour Research and Therapy.

[B12-behavsci-15-00008] Engeln R. (2023). Brains over beauty: A preregistered test of the effects of objectification on women’s cognitive performance. PLoS ONE.

[B13-behavsci-15-00008] Fardouly J., Diedrichs P. C., Vartanian L. R., Halliwell E. (2015). Social comparisons on social media: The impact of Facebook on young women’s body image concerns and mood. Body Image.

[B14-behavsci-15-00008] Feltman C. E., Szymanski D. M. (2018). Instagram Use and Self-Objectification: The Roles of Internalization, Comparison, Appearance Commentary, and Feminism. Sex Roles.

[B15-behavsci-15-00008] Festinger L. (1954). A theory of social comparison processes. Human Relations.

[B16-behavsci-15-00008] Fredrickson B. L., Roberts T. A. (1997). Objectification theory: Toward understanding women’s lived experiences and mental health risks. Psychology of Women Quarterly.

[B17-behavsci-15-00008] Gao J., Feng Y., Xu S., Wilson A., Li H., Wang X., Sun X., Wang Y. (2023). Appearance anxiety and social anxiety: A mediated model of self-compassion. Frontiers in Public Health.

[B18-behavsci-15-00008] Gibbons F. X., Buunk B. P. (1999). Individual differences in social comparison: Development of a scale of social comparison orientation. Journal of Personality and Social Psychology.

[B19-behavsci-15-00008] Gonzales A. L., Hancock J. (2011). Mirror, mirror on my Facebook wall: Effects of Facebook exposure on self-esteem. Cyberpsychology, Behavior, and Social Networking.

[B20-behavsci-15-00008] Gotlib I. H., Joormann J., Minor K. L., Hallmayer J. (2008). HPA axis reactivity: A mechanism underlying the associations among 5-HTTLPR, stress, and depression. Biological Psychiatry.

[B21-behavsci-15-00008] Grabe S., Hyde J. S., Lindberg S. M. (2007). Body objectification and depression in adolescents: The role of gender, shame, and rumination. Psychology of Women Quarterly.

[B22-behavsci-15-00008] Grabe S., Ward L. M., Hyde J. S. (2008). The role of the media in body image concerns among women: A meta-analysis of experimental and correlational studies. Psychological Bulletin.

[B23-behavsci-15-00008] Homan K. J., Tylka T. L. (2015). Self-compassion moderates body comparison and appearance self-worth’s inverse relationship with body appreciation. Body Image.

[B24-behavsci-15-00008] Huizink A., De Rooij S. (2018). Prenatal stress and models explaining risk for psychopathology revisited: Generic vulnerability and divergent pathways. Development and Psychopathology.

[B25-behavsci-15-00008] Karsay K., Knoll J., Matthes J. (2018). Sexualizing media use and self-objectification: A meta-analysis. Psychology of Women Quarterly.

[B26-behavsci-15-00008] Kim H., Markus H. R. (1999). Deviance or uniqueness, harmony or conformity? A cultural analysis. Journal of Personality and Social Psychology.

[B27-behavsci-15-00008] Kim J. W., Chock T. M. (2015). Body image 2.0: Associations between social grooming on Facebook and body image concerns. Computers in Human Behavior.

[B28-behavsci-15-00008] Koopmann-Holm B., Beccari A., Oosthuizen M. (2024). Individual and cultural differences in compassion, noticing suffering, and well-being: Consequences of wanting to avoid feeling negative. Psychology of Compassion.

[B29-behavsci-15-00008] Lazarus R. S., Folkman S. (1984). Stress, Appraisal, and Coping.

[B30-behavsci-15-00008] Leary M. R., Tate E. B., Adams C. E., Batts Allen A., Hancock J. (2007). Self-compassion and reactions to unpleasant self-relevant events: The implications of treating oneself kindly. Journal of Personality and Social Psychology.

[B31-behavsci-15-00008] Levine M. P., Murnen S. K. (2009). “Everybody knows that mass media are/are not [pick one] a cause of eating disorders”: A critical review of evidence for a causal link between media, negative body image, and disordered eating in females. Journal of Social and Clinical Psychology.

[B32-behavsci-15-00008] Luo X. -H., Zou J. -X., Yuan H. -L., Chen X. (2023). Reliability and validity of the Youth Appearance Anxiety Scale. Chinese Journal of Mental Health.

[B33-behavsci-15-00008] McKinley N. M., Hyde J. S. (1996). The objectified body consciousness scale: Development and validation. Psychology of Women Quarterly.

[B34-behavsci-15-00008] Moradi B., Huang Y. P. (2008). Objectification theory and psychology of women: A decade of advances and future directions. Psychology of Women Quarterly.

[B35-behavsci-15-00008] Murnen S. K., Seabrook R. (2012). Feminist Perspectives on Body Image and Physical Appearance. Encyclopedia of Body Image and Human Appearance.

[B36-behavsci-15-00008] Mussweiler T., Strack F., Suls J., Wheeler L. (2000). Consequences of Social Comparison. Handbook of Social Comparison.

[B37-behavsci-15-00008] Neff K. D. (2003). The development and validation of a scale to measure self-compassion. Self and Identity.

[B38-behavsci-15-00008] Neff K. D. (2011). Self-compassion, self-esteem, and well-being. Social and Personality Psychology Compass.

[B39-behavsci-15-00008] Neff K. D., Germer C. K. (2013a). A pilot study and randomized controlled trial of the mindful self-compassion program. Journal of Clinical Psychology.

[B40-behavsci-15-00008] Neff K. D., Germer C. K. (2013b). The mindful self-compassion workbook: A proven way to accept yourself, build inner strength, and thrive.

[B41-behavsci-15-00008] Neff K., Germer C. (2022). The role of self-compassion in psychotherapy. World Psychiatry: Official Journal of the World Psychiatric Association (WPA).

[B42-behavsci-15-00008] Neff K. D., Pisitsungkagarn K., Hsieh Y. (2008). Self-compassion and self-construal in the United States, Thailand, and Taiwan. Journal of Cross-Cultural Psychology.

[B43-behavsci-15-00008] Neff K. D., Pommier E. (2013). The relationship between self-compassion and other-focused concern among college undergraduates, community adults, and practicing meditators. Self and Identity.

[B44-behavsci-15-00008] Noll S. M., Frederickson B. L. (1998). A mediational model of the effects of self-objectification on women’s mental health. Psychology of Women Quarterly.

[B45-behavsci-15-00008] Park J., Kim B., Park S. (2021). Understanding the behavioral consequences of upward social comparison on social networking sites: The mediating role of emotions. Sustainability.

[B46-behavsci-15-00008] Ridgway J. L., Clayton R. B. (2016). Instagram Unfiltered: Exploring Associations of Body Image Satisfaction, Instagram #Selfie Posting, and Negative Romantic Relationship Outcomes. Cyberpsychology, Behavior, and Social Networking.

[B47-behavsci-15-00008] Rollero C., De Piccoli N. (2017). Self-Objectification and Personal Values. An Exploratory Study. Frontiers in psychology.

[B48-behavsci-15-00008] Scully M., Swords L., Nixon E. (2023). Social comparisons on social media: Online appearance-related activity and body dissatisfaction in adolescent girls. Irish Journal of Psychological Medicine.

[B49-behavsci-15-00008] Taylor S. E., Lobel M. (1989). Social comparison activity under threat: Downward evaluations of others and upward dissimilar comparisons. Psychological Review.

[B50-behavsci-15-00008] Tiggemann M., Hayden S., Brown Z., Veldhuis J. (2018). The effect of Instagram “likes” on women’s social comparison and body dissatisfaction. Body Image.

[B51-behavsci-15-00008] Tiggemann M., Slater A. (2014). NetGirls: The internet, Facebook, and body image concern in adolescent girls. International Journal of Eating Disorders.

[B52-behavsci-15-00008] Tiggemann M., Williams E. (2011). The role of self-objectification in disordered eating, depressed mood, and sexual functioning among women: A comprehensive test of objectification theory. Psychology of Women Quarterly.

[B53-behavsci-15-00008] Vogel E. A., Rose J. P., Roberts L. R., Eckles K. (2014). Social comparison, social media, and self-esteem. Psychology of Popular Media Culture.

[B54-behavsci-15-00008] Winn L., Cornelius R. (2020). Self-objectification and cognitive performance: A systematic review of the literature. Frontiers in Psychology.

[B55-behavsci-15-00008] Wong C. C. Y., Mak W. W. S. (2013). Differentiating the role of three self-compassion components in buffering cognitive-personality vulnerability to depression among Chinese in Hong Kong. Journal of Counseling Psychology.

[B56-behavsci-15-00008] Wood J. V. (1989). Theory and research concerning social comparisons of personal attributes. Psychological Bulletin.

[B57-behavsci-15-00008] Wu Y., Xue Y., Zhao X., Han S., Wu W. (2024). Unravelling the veil of appearance anxiety: Exploring social media use among Chinese young people. BMC Psychology.

[B58-behavsci-15-00008] Zubin J., Spring B. (1977). Vulnerability: A new view of schizophrenia. Journal of Abnormal Psychology.

